# Longitudinal trait and state-like differences in the components model of addiction: An illustration through social media addiction and work addiction

**DOI:** 10.1556/2006.2024.00055

**Published:** 2024-10-30

**Authors:** Zsolt Horváth, Bernadette Kun, Orsolya Király, Borbála Paksi, Mark D. Griffiths, Zsolt Demetrovics

**Affiliations:** 1 Institute of Psychology, ELTE Eötvös Loránd University, Budapest, Hungary; 2 Centre of Excellence in Responsible Gaming, University of Gibraltar, Gibraltar, Gibraltar; 3 Institute of Education, ELTE Eötvös Loránd University, Budapest, Hungary; 4 Psychology Department, Nottingham Trent University, Nottingham, UK; 5 College of Education, Psychology and Social Work, Flinders University, Adelaide, Australia

**Keywords:** addictive behaviors, behavioral addictions, problematic social media use, social media addiction, work addiction, workaholism

## Abstract

**Background and aims:**

Conflicting findings have been reported for the longitudinal course of behavioral addictions, especially for social media addiction (SMA) and work addiction (WA). Therefore, evaluating whether these constructs are more trait-like or state-like might be informative. The aim of the present study was to examine the proportion of variance of SMA and WA symptoms (as defined by the components model of addiction) explained by trait and occasion-specific factors in addition to exploring cross-lagged relationships between SMA and WA.

**Methods:**

Young adults from a representative sample who continuously used social media and worked at least 40 hours a week during the first three waves of the Budapest Longitudinal Study were included (*N* = 1,551; Females: 50.6%; Age: *M* = 27.7 years [*SD* = 4.40]). The Bergen Social Media Addiction Scale and the Bergen Work Addiction Scale were administered in all three waves.

**Results:**

A latent state-trait model with a general trait factor was considered for both SMA and WA. Symptomatic variability in SMA was explained approximately equally by trait and state-like factors, while WA-related symptom variability was mostly attributed to state-like factors. SMA negatively predicted WA over time, while WA showed a positive cross-lagged effect on SMA.

**Discussion and Conclusions:**

While the symptoms of WA were more state-like, the trait-like effects were stronger in SMA. Situational influences and previous symptom severities might have to be considered in the screening process.

## Introduction

The 11th revision of the International Classification of Diseases (ICD-11) defines disorders due to addictive behaviors (also known as behavioral addictions [BAs]) as syndromes that are characterized by the repeated presence of a rewarding behavior despite the significant distress and impairment in personal functioning it causes. The ICD-11 includes gaming disorder and gambling disorder in this category (World Health Organization, 2018), while gambling disorder is also included the Diagnostic and Statistical Manual of Mental Disorders (DSM-5) (American Psychiatric Association, 2013).

However, the potentially addictive nature of several other behaviors has also been explored and reported. The conceptualization and operationalization of these BAs is most frequently guided by the components model of addiction (CMA). The CMA suggests that each addictive disorder shares and requires the presence of six core symptoms: salience, mood modification, tolerance, withdrawal, conflict, and relapse (Griffiths, 2005). This framework has facilitated the development of numerous psychometric scales assessing potentially addictive behaviors, including (among others) exercise addiction (Terry, Szabo, & Griffiths, 2004), work addiction (Andreassen, Griffiths, et al., 2012), problematic pornography use (Bőthe et al., 2018), shopping addiction (Andreassen et al., 2015), sex addiction (Andreassen, Pallesen, Griffiths, Torsheim, & Sinha, 2018), and social media addiction (Andreassen, Torsheim, et al., 2012).

The present study examined the CMA specifically through social media addiction (SMA), which is also suggested to be a strong candidate of being a recognized BA (Brand et al., 2020), and work addiction (WA) which has also received much scientific attention. Consequently, both SMA and WA are conceptualized in the present study as BAs:^1^ affected individuals may experience the aforementioned six common addictive symptoms as a result of excessive work and excessive social media use (Andreassen, 2015; Andreassen & Pallesen, 2016). Despite the contribution of the CMA in the field of BAs, it has also been criticized by a few scholars. The main arguments are that (i) the CMA takes an simplistic approach in describing symptoms (e.g., it does not focus on specific symptoms of each BA), (ii) the role of tolerance and withdrawal in BAs is questionable, and (iii) it does not distinguish between central and peripheral symptoms (e.g., relapse vs. salience, respectively) (Billieux, Flayelle, Rumpf, & Stein, 2019), as well as between symptoms and processes underlying BAs (e.g., mood modification). Consequently, BAs defined using the CMA theory may have low clinical utility and diagnostic validity (e.g., it might be questionable whether these constructs are associated with functional impairment and differ from excessive but non-problematic use) (Billieux et al., 2019; Castro-Calvo et al., 2022; Kardefelt-Winther et al., 2017).

The longitudinal course of BAs is particularly critical regarding their phenomenology and clinical relevance. Several theories of BAs implicitly or explicitly consider the chronic nature and long-term functional impairment caused by the behavior as an important diagnostic feature (Gullo, Wood, & Saunders, 2022; Kardefelt-Winther et al., 2017). For example, for both SMA and WA, several longitudinal studies have shown strong associations and small changes over time in symptom severity (Andreassen, Hetland, & Pallesen, 2013; Chang et al., 2022; I.-H. Chen et al., 2020; Hakanen & Peeters, 2015; Koós, Demetrovics, Griffiths, & Bőthe, 2022). These findings support the longitudinal stability and persistence of SMA and WA. These findings are linked to the significance of personality traits in both WA and SMA, as these stable dimensions contribute to the long-term persistence of addictive behaviors. Notably, in the case of WA, it is essential to emphasize factors such as low global self-esteem, high perfectionism, negative affectivity, compulsiveness, and anxiety (Kun, Takacs, Richman, Griffiths, & Demetrovics, 2021). In the context of SMA, significant personality factors include higher neuroticism and narcissism, along with lower self-esteem and lower conscientiousness (Huang, 2022; Kircaburun, Demetrovics, & Tosuntaş, 2019). In contrast, other studies have found only modest longitudinal stability for SMA and WA (Raudsepp, 2019; Tóth-Király, Morin, & Salmela-Aro, 2021). Similarly, the transient nature of BAs and the possible role of natural recovery have been indicated by some findings (Konkolÿ Thege, Woodin, Hodgins, & Williams, 2015). There might be greater fluctuations in symptom severity (e.g., rapid progression followed by spontaneous decline in symptom severity) at specific life stages (e.g., young adulthood) and due to situational factors (e.g., acute stress, changes in job demands or organizational settings) (Atroszko, Demetrovics, & Griffiths, 2019; Derevensky, 2019).

Building on these conflicting findings, the latent state-trait theory can offer an alternative and novel perspective for explaining the longitudinal course of BAs, and specifically for SMA and WA defined under the CMA. The latent state-trait theory has been proposed for a variety of psychopathologies to evaluate the extent to which a given condition can be classified as trait-like vs. state-like (Geiser & Lockhart, 2012; Struijs et al., 2020). Latent state-trait models assume that both trait and occasion-specific factors explain the variability of symptom scores over time. On the one hand, a trait factor can be understood as a temporally stable component indicating a general vulnerability for the given BA. On the other, occasion-specific factors illustrate a temporally varying/situation-specific vulnerability for the given BA (Conway, Hipwell, & Stepp, 2017, 2018; Geiser, Hintz, Burns, & Servera, 2021; Geiser & Lockhart, 2012). Although trait and occasion-specific factors have already been distinguished in SMA (Chang et al., 2022; I.-H. Chen et al., 2022; Di Blasi et al., 2022), no research has been conducted to systematically examine these factors' explanatory role in the longitudinal development of symptom scores for CMA-based constructs. Such an approach could help to determine the extent to which SMA and WA (defined by the CMA) are trait-like versus state-like constructs. In line with this, the chronic or transient nature of BAs could be supported by the higher explained variance by temporally stable/trait factors or temporally varying/occasion-specific factors, respectively (Gullo et al., 2022; Konkolÿ Thege et al., 2015).

Distinguishing temporally stable/trait and temporally varying/occasion-specific factors may also contribute to a more nuanced and specific investigation of the longitudinal relationships between BAs (Hamaker, Kuiper, & Grasman, 2015). More specifically, related to the aims of the present study, an association between SMA and WA might be expected at two different levels. First, the association between trait factors may suggest that shared etiological mechanisms and symptomatic components are presented for both SMA and WA (Brand, Young, Laier, Wölfling, & Potenza, 2016; Griffiths, 2005). In these cases, common personality traits, such as self-esteem and negative affectivity, may account for the co-occurrence, but there may also be shared motivational factors. Neither case is linked to intrinsic motives or positive emotions; instead, they are associated with the avoidance of negative emotions and the ‘fear of missing out’ (Schivinski et al., 2020; Shen, Zhang, & Xin, 2022; Van Beek, Hu, Schaufeli, Taris, & Schreurs, 2012).

Second, the interplay of working and social networking activities may contribute to a mutually reinforcing longitudinal association between SMA and WA. For example, cross-lagged analysis in a previous study showed that compulsive internet use longitudinally predicted WA (Quinones, Griffiths, & Kakabadse, 2016). Although SMA and compulsive internet use do not fully overlap, this finding may suggest that SMA contributes to increased WA over time. Conversely, it could also be possible that WA predicts an exacerbation of SMA over time. These hypothesized (although not yet tested) longitudinal mechanisms may be explained by the circumstance that work and social media use are now closely connected in various professional contexts. Individuals often use social media for work-related tasks, including posting and sharing, as well as for building professional relationships and accessing information.

While not extensively studied, individuals affected by WA may also experience a ‘fear of missing out', driving them to constantly strive for maximum performance to avoid missing important opportunities, information, or events. Furthermore, there is evidence that individuals with WA tend to use their smartphones more frequently (Cheung, Lun, & Wang, 2022; Spagnoli, Fabbri, & Barbato, 2019). While this increased smartphone use often relates to work-related activities, it may also hinder their ability to disconnect from the phone, affecting other functions such as the use of social media sites. Overall, the longitudinal association between SMA and WA has not been investigated to date. Therefore, further research is needed in this regard (e.g., by simultaneously considering temporally stable/trait and temporally varying/occasion-specific factors of SMA and WA).

Considering the aforementioned literature, the present exploratory study had two main objectives. First, to examine the longitudinal structural model of the CMA via SMA and WA to determine the proportion of variance in symptom scores explained by temporally stable/trait and temporally varying/occasion-specific factors. The second objective was to investigate the longitudinal relationship between WA and SMA, specifically focusing on the association between trait factors and the cross-lagged relationships between state-like factors of SMA and WA.

## Methods

### Participants and procedure

The present study analyzed representative data from the first three waves of the Budapest Longitudinal Study (BLS) (Horváth et al., 2023; Paksi & Demetrovics, 2021). Sampling was random and stratified by age group and district of residence. The target population was young adults living in Budapest (the capital of Hungary), born between 1984 and 2000. Approximately one year elapsed between consecutive data collections, starting in 2019. Participants were required to provide responses through personal interviews. These combined face-to-face interviews (e.g., sociodemographic data, screening questions on BAs) and self-administered, paper-pencil questionnaires (e.g., symptoms of BAs, psychological scales). In Waves 2 and 3, participants could participate online through computer-assisted web interviews (see Supplementary Fig. S1 for more information).

A total of 3,914 participants completed the survey in at least one wave of the BLS, and 2,563 participants completed the survey in all three waves (females: *N* = 1,302 [50.81%]; mean age at Wave 1: 27.05 years [*SD* = 4.77]). The final sample included 1,551 participants (females: *N* = 784 [50.55%]; mean age at Wave 1: 27.65 years [*SD* = 4.40]). To be eligible for the final sample, it was necessary in all three waves to (i) participate, (ii) use social media on both an average weekday and weekend day in the last 30 days, and (iii) work at least 40 h in an average week (see Supplementary Fig. S1 for the number of participants fulfilling and not fulfilling each of these criteria). Those in the final sample most often used social media for 1–2 h on both an average weekday and weekend day across all three waves. The average working time ranged between 41.63 and 42.12 h in an average week across the waves (Supplementary Table S1).

### Measures

SMA and WA were assessed using the six-item Bergen Social Media Addiction Scale (BSMAS) and the seven-item Bergen Work Addiction Scale (BWAS), respectively (Original versions: Andreassen et al., 2013; Andreassen, Torsheim, et al., 2012; Hungarian versions: Bányai et al., 2017; Orosz, Dombi, Andreassen, Griffiths, & Demetrovics, 2016). The items in both scales cover the six core symptoms described by the CMA. In addition, for the BWAS, an additional symptom assesses work-related health problems. Both scales use a five-point response scale (for the BSMAS: 1 = Never, 5 = Very often; for the BWAS: 0 = Not at all typical, 4 = Very typical). For descriptive statistical analyses, total scale scores and prevalence of risk for SMA and WA were calculated based on polythetic (i.e., responses ‘often’ or ‘almost always’ for the BSMAS and ‘more’ or ‘very typical’ for the BWAS given to at least four symptoms) and monothetic scoring (i.e., similar responses given to all six symptoms). High internal consistencies were obtained for both scales across the three waves (Table 1).

**Table 1.** Pairwise correlations and internal consistencies

	1.	2.	3.	4.	5.	6.
1. Social media addiction – Wave 1	–					
2. Social media addiction – Wave 2	0.27	–				
3. Social media addiction – Wave 3	0.24	0.33	–			
4. Work addiction – Wave 1	0.45	0.20	0.23	–		
5. Work addiction – Wave 2	0.25	0.41	0.28	0.28	–	
6. Work addiction – Wave 3	0.20	0.22	0.47	0.24	0.31	–
Cronbach's *α*	0.91	0.90	0.92	0.85	0.82	0.95

*Notes*. Correlations in the table are Spearman's rho values. All correlations were significant at *p* < 0.001 level (two-tailed).

The frequency of social media use in the past 30 days was measured by separate questions for weekdays and weekends. Participants included in the present study provided responses to these questions along a six-point scale in each wave (1 = Less than an hour, 6 = More than 8 h). In addition, an open-ended question in each wave required participants to indicate how many hours they had spent working in an average week in the past year.

### Statistical analyses

First, descriptive statistical analyses were performed. This involved estimating the prevalence of risk for SMA and WA, and Spearman correlations between the non-normally distributed BSMAS and BWAS total scores across the three waves. In addition, repeated-measures analysis of variance (ANOVA) was used to examine the changes in SMA and WA between Waves 1 and 3.

To address the first research objective, the fit of different latent state-trait models was examined separately for SMA and WA. As a preliminary step, four consecutive and gradually more restrictive levels of longitudinal invariance (i.e., configural, metric, scalar, residual invariance) were examined and compared for the one-factor models of the BSMAS and BWAS (see Supplementary Table S2 for more information) (Liu et al., 2017). The thresholds and residuals of the observed indicators and the factor loadings on the state latent residual factors (i.e., temporally varying/occasion-specific factors accounting for the covariation between symptoms/indicators at a given measurement point; therefore, indicating situational or person-situation interactional effects) were estimated in the latent state-trait models in accordance with the accepted invariance models. Three latent state-trait models were estimated separately for SMA and WA (see Supplementary Figs S2–S7 for full details) (Geiser & Lockhart, 2012).

First, a model with one general trait factor (i.e., a temporally invariant/stable and non-symptom-specific factor accounting for the covariation between all symptoms/indicators over time) and without indicator-specific method factors (i.e., temporally invariant/stable and symptom-specific factors accounting for the covariation between the same, repeatedly measured symptoms over time). Second, a model with correlated indicator-specific trait factors (in the number of items comprising the scales). Third, a model with one general trait factor and with one less correlated method factors than the number of items comprising the scales. In all models, autoregressive effects were estimated between the latent state residual factors (Courvoisier, Eid, Lischetzke, & Schreiber, 2010; Geiser et al., 2021). Measures of reliability (i.e., the proportion of true score variance in observed scores), consistency (i.e., the proportion of temporally stable/trait effects in true score variance) and occasion-specificity (i.e., the proportion of temporally varying/state-like effects in true score variance) were calculated related to the BSMAS and BWAS items (Geiser, 2021).

Regarding the second research objective, a cross-lagged model between SMA and WA was defined. The latent state-trait models accepted for SMA and WA were used to define the temporally stable/trait and occasion-specific, state residual factors in the model. The model included autoregressive and cross-lagged effects between the temporally consecutive SMA and WA state-residual factors. In addition, correlations were estimated between SMA and WA trait factors and between concurrent SMA and WA state residual factors for the same wave.

The invariance, latent state-trait and cross-lagged models were estimated using the weighted least squares means and variances adjusted (WLSMV) procedure and categorical observed indicators (due to the non-normal distribution of the BSMAS and BWAS items). Adequate model fit was demonstrated at values ≥ 0.90 for the comparative fit index (CFI) and Tucker-Lewis index (TLI), and at ≤0.08 for the root mean square error of approximation (RMSEA). Optimal fit was indicated by values ≥ 0.95 for the CFI and TLI and ≤0.05 for the RMSEA. To accept a more restrictive level of invariance, the deterioration in fit between consecutive models should have been ≤0.01 for the CFI and ≤0.015 for the RMSEA (F. F. Chen, 2007).

Descriptive statistics were calculated using IBM SPSS 22.0, while Mplus 8.0 (Muthén & Muthén, 2017) was used for further analyses. Analyses were computed by applying multidimensional longitudinal weighting. This provided a correction for socio-demographic factors associated with sample attrition (i.e., being female, not having a primary level of educational attainment, and being religious were negatively linked to attrition), to ensure a proportional distribution of the sample by residence and age for the young adult population in Budapest. In addition, sensitivity analyses indicated that there were only small differences in SMA and WA between participants who were included and excluded from the final sample. Those who were excluded showed significantly higher scores in Wave 1, whereas participants in the final sample showed higher scores in the other waves in the significant comparisons (Supplementary Table S3).

### Ethics

The Research and Ethical Committee of the Hungarian Medical Research Council approved the protocol of the BLS. All research procedures were performed in accordance with the Declarations of Helsinki. Informed consent was obtained from all participants.

## Results

### Descriptive statistics

The risk for SMA varied between 0.89% and 1.12%, and between 0.00% and 0.14% based on the polythetic and monothetic approaches, respectively. The risk for WA varied between 3.34% and 5.68%, and between 0.23% and 2.84% based on the polythetic and monothetic approaches, respectively (Supplementary Tables S4–S5). Both between SMA scores and between WA scores, there were significant, positive, and weak-to-medium correlations across the three waves. SMA and WA also correlated significantly and positively with each other: medium-strength correlations were observed within the same wave, while weak-strength correlations were found across different waves (Table 1). Significant but only small changes were observed for SMA and WA between Waves 1 and 3. The highest average severity for SMA was present in Wave 2, while for WA it was in Wave 3 – in both cases, with significantly higher values than in the other waves (Table 2).

**Table 2.** Changes in social media addiction and work addiction between Waves 1 and 3

	Social media addiction	Work addiction
Wave 1 M (SD)	7.81 (3.17)	4.12 (4.60)
Wave 2 M (SD)	8.61 (3.51)	4.32 (4.77)
Wave 3 M (SD)	8.05 (3.35)	5.39 (6.59)
F	38.85***	38.62***
Partial *η*^*2*^	0.02	0.02
Post-hoc test: Wave 1 vs. Wave 2	*d* = 0.21***	*d* = 0.04
Post-hoc test: Wave 1 vs. Wave 3	*d* = 0.06*	*d* = 0.22***
Post-hoc test: Wave 2 vs. Wave 3	*d* = 0.14***	*d* = 0.19***

*Notes*: M (SD): mean (standard deviation). F: F-statistic related to repeated-measures ANOVA. Partial *η*^*2*^: partial eta-squared. *d*: Cohen's *d.* Bonferroni correction was used for post-hoc tests. Level of significance (two-tailed): *p < 0.050; **p < 0.010; ***p < 0.001.

### Latent state-trait models

Residual and scalar invariance levels were reached related to the longitudinal one-factor models of BSMAS and BWAS, respectively. Both invariance models were characterized by adequate-optimal levels of model fit (Supplementary Table S6). Therefore, these longitudinal invariance models were the basis of the latent state-trait models tested. For both SMA and WA, the latent state-trait model with one general trait factor and without method factors showed adequate-optimal model fit. In both cases, this latent state-trait model was preferred and interpreted because the validity of the other two latent state-trait models was questionable due to statistical problems. In these cases, the latent variable covariance matrices were not positively definitive, despite the appropriate theoretical model specification (Supplementary Figs S3–S4 and S6–S7). Haywood cases were identified on the latent variables, resulting in biased, out-of-range correlations (i.e., greater than 1). These statistically invalid solutions were therefore rejected and not interpreted (see Supplementary Table S6).

For SMA, all symptoms showed significant, positive, and strong factor loadings on the SMA general trait factor as well as on the wave-specific latent state residual factors (the only exception was salience with a moderately strong loading in Wave 2). A non-significant autoregressive effect was observed between Waves 1 and 2, while a significant, negative, and moderate autoregressive effect was shown between Waves 2 and 3. Except for salience, higher proportions of the true score variance for each SMA symptom in Waves 1 and 3 were explained by temporally varying/occasion-specific effects (52%–64% and 56%–68% in Waves 1 and 3, respectively). In contrast, the true score variance in salience in these waves was explained by the temporally stable/trait-like factor to a slightly greater extent (56% and 51% in Waves 1 and 3, respectively). In Wave 2, higher proportions of the true score variance of each symptom were explained by the temporally stable/trait factor (53%–71%). Overall, in all three waves, withdrawal and conflict in SMA were characterized by the highest proportion of temporally varying/occasion-specific effects, while the temporally stable/trait-like effect was the highest for salience in SMA (Table 3).

**Table 3.** Factor loadings, reliability estimates and autoregressive effects in the latent state-trait model with one trait factor and without method factors related to social media addiction (SMA)

	Latent state residual factor – W1 *λ* (SE)	Latent state residual factor – W2 *λ* (SE)	Latent state residual factor – W3 *λ* (SE)	General trait factor *λ* (SE)	Reliability (SE)	Consistency (SE)	Consistency %	Occasion-specificity (SE)	Occasion-specificity %
Salience – W1	0.59 (0.03)***			0.66 (0.02)***	0.77 (0.01)***	0.43 (0.03)***	56%	0.34 (0.03)***	44%
Tolerance – W1	0.64 (0.03)***			0.62 (0.02)***	0.80 (0.01)***	0.39 (0.03)***	48%	0.41 (0.04)***	52%
Mood modification – W1	0.67 (0.03)***			0.60 (0.02)***	0.81 (0.01)***	0.36 (0.03)***	45%	0.45 (0.04)***	55%
Relapse – W1	0.65 (0.03)***			0.62 (0.02)***	0.80 (0.01)***	0.38 (0.03)***	47%	0.42 (0.03)***	53%
Withdrawal – W1	0.74 (0.03)***			0.55 (0.03)***	0.84 (0.01)***	0.30 (0.03)***	36%	0.54 (0.04)***	64%
Conflict – W1	0.73 (0.03)***			0.56 (0.03)***	0.84 (0.01)***	0.31 (0.03)***	37%	0.53 (0.04)***	63%
Salience – W2		0.48 (0.04)***		0.71 (0.02)***	0.73 (0.01)***	0.52 (0.04)***	71%	0.21 (0.04)***	29%
Tolerance – W2		0.53 (0.04)***		0.69 (0.03)***	0.75 (0.01)***	0.48 (0.04)***	65%	0.26 (0.05)***	35%
Mood modification – W2		0.56 (0.04)***		0.67 (0.03)***	0.76 (0.01)***	0.47 (0.05)***	62%	0.29 (0.06)***	39%
Relapse – W2		0.54 (0.04)***		0.68 (0.03)***	0.75 (0.01)***	0.48 (0.04)***	64%	0.27 (0.05)***	36%
Withdrawal – W2		0.63 (0.04)***		0.63 (0.04)***	0.78 (0.01)***	0.41 (0.05)***	53%	0.37 (0.07)***	47%
Conflict – W2		0.62 (0.04)***		0.63 (0.03)***	0.78 (0.01)***	0.42 (0.05)***	54%	0.36 (0.07)***	46%
Salience – W3			0.62 (0.02)***	0.64 (0.02)***	0.79 (0.01)***	0.40 (0.02)***	51%	0.39 (0.03)***	49%
Tolerance – W3			0.68 (0.02)***	0.60 (0.02)***	0.81 (0.01)***	0.36 (0.02)***	44%	0.46 (0.03)***	56%
Mood modification – W3			0.71 (0.02)***	0.58 (0.02)***	0.83 (0.01)***	0.33 (0.02)***	40%	0.50 (0.03)***	60%
Relapse – W3			0.69 (0.02)***	0.59 (0.02)***	0.82 (0.01)***	0.35 (0.02)***	43%	0.47 (0.03)***	57%
Withdrawal – W3			0.77 (0.02)***	0.52 (0.02)***	0.86 (0.01)***	0.27 (0.02)***	32%	0.59 (0.03)***	68%
Conflict – W3			0.76 (0.02)***	0.53 (0.02)***	0.85 (0.01)***	0.28 (0.03)***	33%	0.58 (0.03)***	68%
Autoregressive effect: W1 → W2 (*β* [SE])	−0.04 (0.07)								
Autoregressive effect: W2 → W3 (*β* [SE])	−0.32 (0.10)**								

*Notes*. Model fit: *χ*^*2*^ (188) = 1011.248; *p* < 0.001. CFI = 0.980. TLI = 0.984. RMSEA [90% CI] = 0.051 [0.048; 0.054]. W1: Wave 1. W2: Wave 2. W3: Wave 3. *λ*: standardized factor loading. *β*: standardized regression coefficient. SE: standard error. Level of significance (two-tailed): *p < 0.050; **p < 0.010; ***p < 0.001.

For WA, significant, positive, and strong factor loadings were obtained on all wave-specific latent state residual factors, while all symptoms showed non-significant loadings on the WA general trait factor. Significant, positive, medium-strong autoregressive effects were present between consecutive waves. Consistent with the pattern observed in the factor loadings, the true score variance of WA symptoms was predominantly explained by temporally varying/occasion-specific effects (90%–98%). However, it is important to note that low reliability indices (<0.70) were present for multiple symptoms. Only the conflict and the problems symptoms showed at least adequate reliability across the three waves (Table 4).

**Table 4.** Factor loadings, reliability estimates and autoregressive effects in the latent state-trait model with one trait factor and without method factors related to work addiction (WA)

	Latent state residual factor – W1 *λ* (SE)	Latent state residual factor – W2 *λ* (SE)	Latent state residual factor – W3 *λ* (SE)	General trait factor *λ* (SE)	Reliability (SE)	Consistency (SE)	Consistency %	Occasion-specificity (SE)	Occasion-specificity %
Salience – W1	0.77 (0.02)***			0.16 (0.09)	0.62 (0.02)***	0.03 (0.03)	4%	0.60 (0.04)***	96%
Tolerance – W1	0.60 (0.03)***			0.20 (0.11)	0.40 (0.03)***	0.04 (0.04)	10%	0.36 (0.04)***	90%
Mood modification – W1	0.72 (0.03)***			0.17 (0.10)	0.55 (0.02)***	0.03 (0.03)	5%	0.52 (0.04)***	95%
Relapse – W1	0.80 (0.02)***			0.15 (0.08)	0.66 (0.02)***	0.02 (0.03)	3%	0.64 (0.03)***	97%
Withdrawal – W1	0.81 (0.02)***			0.15 (0.08)	0.67 (0.02)***	0.02 (0.02)	3%	0.65 (0.03)***	97%
Conflict – W1	0.82 (0.02)***			0.14 (0.08)	0.70 (0.02)***	0.02 (0.02)	3%	0.68 (0.03)***	97%
Problems – W1	0.85 (0.02)***			0.13 (0.07)	0.74 (0.02)***	0.02 (0.02)	2%	0.72 (0.03)***	98%
Salience – W2		0.76 (0.02)***		0.12 (0.07)	0.51 (0.03)***	0.02 (0.02)	3%	0.49 (0.03)***	97%
Tolerance – W2		0.56 (0.03)***		0.14 (0.08)	0.27 (0.02)***	0.02 (0.02)	8%	0.24 (0.02)***	92%
Mood modification – W2		0.84 (0.02)***		0.15 (0.09)	0.65 (0.03)***	0.03 (0.03)	4%	0.63 (0.04)***	96%
Relapse – W2		0.82 (0.02)***		0.12 (0.07)	0.61 (0.03)***	0.02 (0.02)	3%	0.59 (0.03)***	97%
Withdrawal – W2		0.64 (0.02)***		0.09 (0.05)	0.34 (0.02)***	0.01 (0.01)	3%	0.33 (0.02)***	97%
Conflict – W2		0.90 (0.01)***		0.12 (0.07)	0.76 (0.02)***	0.02 (0.02)	2%	0.74 (0.03)***	98%
Problems – W2		0.92 (0.01)***		0.11 (0.06)	0.82 (0.02)***	0.02 (0.02)	2%	0.80 (0.03)***	98%
Salience – W3			0.87 (0.01)***	0.13 (0.07)	0.74 (0.02)***	0.02 (0.02)	3%	0.72 (0.03)***	97%
Tolerance – W3			0.81 (0.03)***	0.20 (0.11)	0.64 (0.03)***	0.05 (0.05)	7%	0.59 (0.04)***	93%
Mood modification – W3			0.84 (0.02)***	0.15 (0.08)	0.68 (0.02)***	0.03 (0.03)	4%	0.66 (0.03)***	96%
Relapse – W3			0.90 (0.01)***	0.13 (0.07)	0.78 (0.02)***	0.02 (0.02)	2%	0.77 (0.02)***	98%
Withdrawal – W3			0.92 (0.01)***	0.13 (0.07)	0.84 (0.01)***	0.02 (0.02)	2%	0.82 (0.02)***	98%
Conflict – W3			0.94 (0.01)***	0.12 (0.07)	0.88 (0.01)***	0.02 (0.02)	2%	0.87 (0.02)***	98%
Problems – W3			0.93 (0.01)***	0.11 (0.06)	0.86 (0.01)***	0.01 (0.02)	2%	0.84 (0.02)***	98%
Autoregressive effect: W1 → W2 (*β* [SE])	0.46 (0.03)***								
Autoregressive effect: W2 → W3 (*β* [SE])	0.54 (0.03)***								

*Notes*. Model fit: *χ*^*2*^ (238) = 1773.159; *p* < 0.001. CFI = 0.975. TLI = 0.978. RMSEA [90% CI] = 0.062 [0.059; 0.065]. W1: Wave 1. W2: Wave 2. W3: Wave 3. *λ*: standardized factor loading. *β*: standardized regression coefficient. SE: standard error. Level of significance (two-tailed): *p < 0.050; **p < 0.010; ***p < 0.001.

### Cross-lagged analysis

**Fig. 1.**
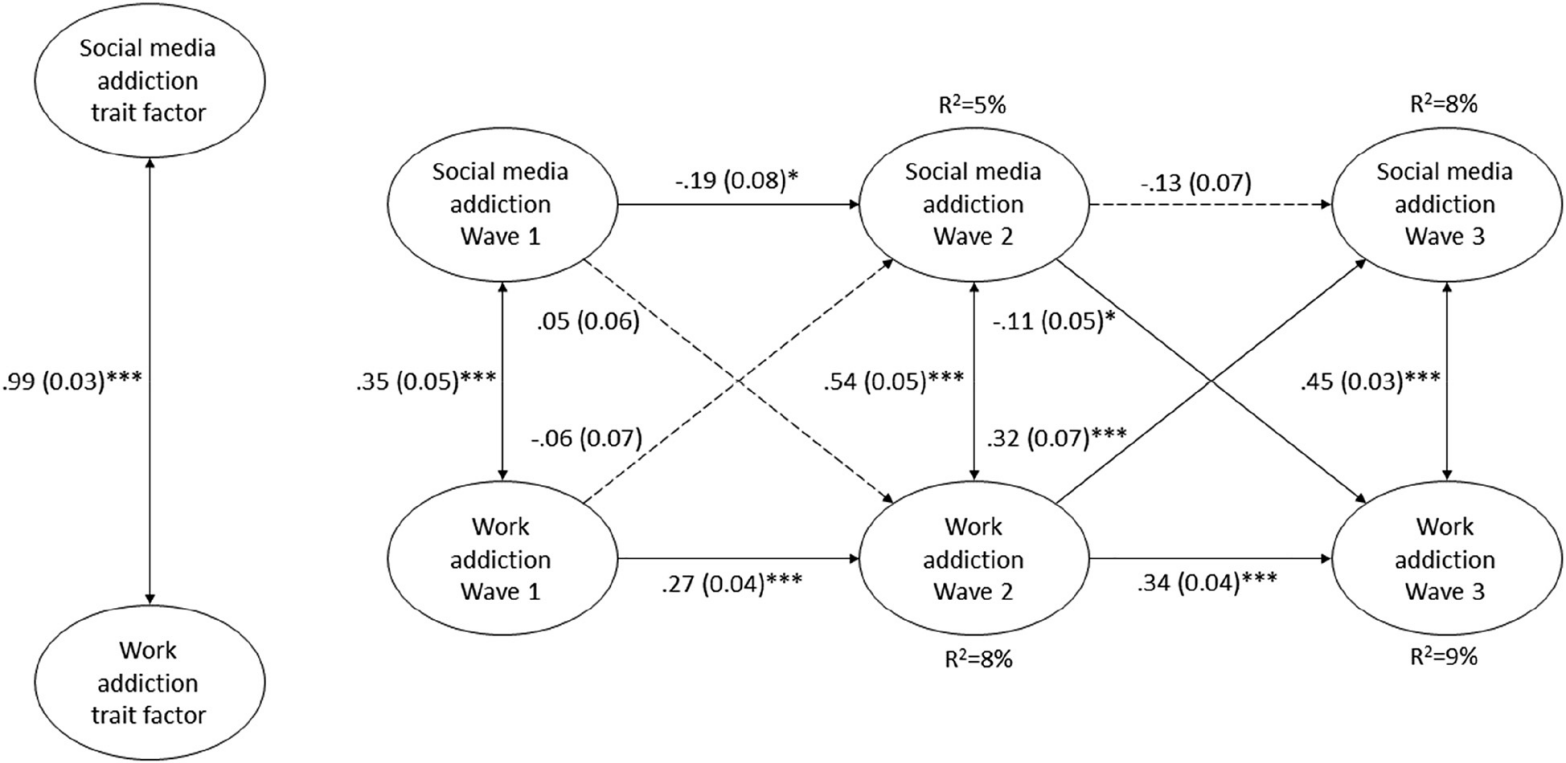
Cross-lagged model between state residual factors of social media addiction (SMA) and work addiction (WA). Model fit: *χ*^*2*^ (797) = 3697.182; *p* < 0.001. CFI = 0.967. TLI = 0.969. RMSEA [90% CI] = 0.047 [0.045; 0.048]. Values on single-headed arrows are standardized regression coefficients (β) and corresponding standard errors (SE). Values on double-headed arrows are correlations (*r*) and corresponding standard errors (SE). Level of significance (two-tailed): *p < 0.05; **p < 0.01; ***p < 0.001

The cross-lagged model between SMA and WA showed optimal model fit (Fig. 1). Significant and positive correlations were observed between the SMA and WA factors. On the one hand, there was a very strong correlation between the trait factors. On the other hand, medium-strong correlations between concurrent state residual factors were also demonstrated (Fig. 1).

There were significant, positive, and weak-medium autoregressive effects between consecutive state residual factors of WA. That is, increased WA at given measurement predicted elevated WA at the subsequent measurement. For SMA, there was only a significant autoregressive effect between Waves 1 and 2, indicating a negative and weak relationship. This negative autoregressive effect implies that increased SMA in Wave 1 contributed to decreased SMA in Wave 2 (Fig. 1).

Two significant cross-lagged relationships were detected. SMA in Wave 2 significantly, negatively, and weakly predicted WA in Wave 3. That is, higher levels of SMA in Wave 2 predicted lower rates of WA in Wave 3. In contrast, WA in Wave 2 predicted SMA in Wave 3 significantly, positively and with moderate strength. This means that increased WA in Wave 2 contributed to increased SMA in Wave 3 (Fig. 1).

## Discussion

The present study explored the longitudinal course of SMA and WA over an approximately 2-year period in a representative sample of young adults from a novel perspective. The findings of the present study advance the understanding of the degree to which BAs defined by the CMA (specifically, SMA and WA) can be considered trait vs. state-like constructs. The findings indicated that there may be considerable differences in the longitudinal structure of different BAs, even though they were defined by similar symptoms according to the CMA. However, it is important to draw cautious conclusions on the CMA in general, because only SMA and WA were investigated. It is possible that different longitudinal patterns would have been found for other BAs defined by the CMA.

### Latent state-trait models of social media and work addiction

The first aim of the present study was to examine the longitudinal structural model of the CMA via SMA and WA to determine the proportion of variance in symptom scores explained by temporally stable/trait and temporally varying/occasion-specific factors. For both SMA and WA, a model with one general trait factor and three state residual factors was accepted. The general trait factor can be interpreted as a temporally stable and non-symptom-specific effect (i.e., accounting for the covariation between all symptoms over time), indicating a general vulnerability for SMA and WA. The state residual factors indicated a temporally varying and situation-specific vulnerability for the given BA (i.e., accounting for the covariation between symptoms at a given measurement point). To the best of the authors' knowledge, the present study is the first to systematically assess how these temporally stable and varying effects simultaneously explain the longitudinal variability of symptoms in BAs.

For SMA, a slightly higher proportion of symptom variance was attributed to temporally varying/occasion-specific effects. However, to an approximately similar extent, variability in symptoms was also attributed to a trait factor expressing a general and temporally stable SMA vulnerability. The relatively high proportion of the latter may possibly explain previous research findings that reported longitudinally high correlations and small changes in SMA symptom severity (Chang et al., 2022; I.-H. Chen et al., 2020; Koós et al., 2022). Furthermore, in SMA, there were differences in symptoms as to whether they were more trait-like or state-like. Longitudinal variation in salience symptom scores was explained to a greater extent by the temporally stable/trait factor, whereas withdrawal and conflict symptoms were more sensitive to changes in state-like situational influences.

Critiques have been raised regarding the clinical and diagnostic validity of salience and withdrawal (Billieux et al., 2019; Karriker-Jaffe, Klinger, Witbrodt, & Kaskutas, 2018). and, therefore, these results may provide additional information on these components. The widespread availability and frequent use of social media may explain why salience/preoccupation may be more of a trait-like symptom. Furthermore, one possible explanation is that SMA is linked to increased compulsiveness (Akça et al., 2019). This compulsion is characterized by cravings and preoccupation, with social media as the focal point. Additionally, SMA is correlated with heightened emotional instability (Marengo, Poletti, & Settanni, 2020), which can further exacerbate an individual's ‘fear of missing out’ (Rozgonjuk, Sindermann, Elhai, & Montag, 2021). This phenomenon underscores a distinct connection with SMA (Casale, Rugai, & Fioravanti, 2018). In addition, the more state-like course of withdrawal symptoms may be due to increased fluctuations of related psychological states (e.g., becoming restless) over time. Therefore, it might be possible that withdrawal alone may be less able to capture the more severe and compulsive stages of SMA, and the role of other components should be considered as well (e.g., tolerance, coping) (Gullo et al., 2022; Sassover & Weinstein, 2020). Withdrawal and conflict may exhibit state-like effects because they are strongly affected by external factors, including other individuals and external circumstances. These factors can determine whether an individual can use social media sites and depends on the prevalence of social media use in their environment and the extent to which it conflicts with their usage habits.

In contrast, WA was more likely to be considered a predominantly state-like construct, and the temporally stable/trait factor explained only a negligible amount of variance in symptoms. The high explanatory levels of the temporally varying/occasion-specific factors highlight the relevance of episodic or situational influences in the development of WA symptoms over time. Episodic longitudinal patterns were also observed for other BAs in previous research (Konkolÿ Thege et al., 2015). This result corroborates the findings of meta-analyses, which indicate that personality traits have a relatively small effect size in the development of WA, explaining only a small proportion of it (Clark, Michel, Zhdanova, Pui, & Baltes, 2016; Kun et al., 2021).

The present longitudinal study underscores the need to shift the focus towards macro-level and meso-level factors in understanding WA (Atroszko et al., 2019; Tóth-Király, Bőthe, & Orosz, 2018), where situational effects play a more significant role in the problem. When considering macro-factors (i.e., culture, society, economy), it becomes evident that the prevalence of WA varies substantially by region; for example, it is much higher in Far Eastern countries (39.8%) compared to Europe (where the prevalence ranges from 7% to 10%) (Andreassen, Nielsen, Pallesen, & Gjerstad, 2019; Kang, 2020; Kun, Magi, Felvinczi, Demetrovics, & Paksi, 2020). Regarding meso-level factors, it is imperative to give greater attention to the organizational climate, workplace values, expectations, conditions, and norms. Finally, it is important to consider that the work-related inclusion criterion (i.e., work at least 40 h in an average week during the entire study period) may also have influenced and biased the findings related to WA. Because the data collection occurred during the COVID-19 pandemic, it is possible that some of the excluded participants experienced negative effects on their employment (e.g., employer-ordered reduction in working time, loss of job), in addition to socioeconomical differences that explained the continuation of working at least 40 h on average in this period (e.g., individuals with higher education levels, working in the office).

The role of autoregressive effects was another significant difference between SMA and WA. For WA, symptom severity at a given wave had a positive carryover effect on subsequent symptom severity. Overall, longitudinal risk in WA may be more attributable to a prior elevated WA symptom severity than to a general and temporally stable WA vulnerability factor. In essence, while the role of the stable trait factors in the long-term persistence of WA is relatively small, episodic-specific or situation-specific increase in WA severity can pose a significant risk for the longitudinal development and stability. Several explanations can shed light on this phenomenon. WA is driven not so much by short-term rewards but by long-term reinforcements like recognition, respect, promotion, and higher positions (Ng, Sorensen, & Feldman, 2007). Achieving these rewards requires prolonged and persistent efforts. Moreover, individuals may not always have the option to change jobs whenever they want, so if an organization maintains harmful characteristics, such as destructive competition, excessive or conflicting expectations, or supervisors who promote a culture with a risk for WA (Ng et al., 2007; Porter, 2001), it can keep individuals trapped by WA over the long term. Lastly, it is essential to consider the context of the period under study, which coincided with the COVID-19 pandemic. This external factor was reported to have exacerbated symptoms of excessive and compulsive work (Shkoler et al., 2021). Economic insecurity, a surge in unemployment, the global shift to online work, and an increased imbalance in work-life dynamics (Trógolo, Moretti, & Medrano, 2022) could have pushed those already affected by work addiction further into compulsive working.

In contrast, a negative autoregressive relationship was seen between waves for SMA. This pattern may represent a natural maturing out and symptom relief observed previously for BAs (Konkolÿ Thege et al., 2015). It is conceivable that age-related changes also play a role in this phenomenon. Research indicates that SMA is more prevalent among younger individuals (Cheng, Lau, Chan, & Luk, 2021). Consequently, the individuals in the sample exhibited less compulsive social media use as they grew older. Furthermore, the potential impact of the COVID-19 pandemic must again be considered. The third wave of the data collection occurred after the lifting of complete lockdowns, with nearly half of the Hungarian population (approximately 5 million individuals), having received vaccinations. As a result, individuals had more opportunities for in-person interactions, meetings, and social activities, reducing their reliance on online spaces. It is plausible that individuals who were compelled to use social media during the pandemic, as it was the primary means of staying in touch, but preferred face-to-face relationships, returned to in-person activities (Koós et al., 2022). Moreover, the results cannot be isolated from the methodological characteristics of the study. Multiple factors may have contributed to the increased temporally varying/occasion-specific influences on SMA and WA. The sample consisted of young adults and mostly individuals with low symptom severity and without risk, which may have contributed to the higher state-like fluctuations in symptoms over time (Castro-Calvo et al., 2022; Derevensky, 2019).

### Longitudinal relationship between social media and work addiction

The second aim of the present study was to investigate the longitudinal relationship between WA and SMA at different levels of these constructs. The distinction between temporally stable/trait and temporally varying/occasion-specific factors in SMA and WA also allowed more precise exploration of the longitudinal relationship between them. More specifically, positive correlations were observed between trait factors and between occasion-specific factors in the same wave, while both positive and negative cross-lagged associations were found between SMA and WA. The relevance of these results lies in that, to the best of the authors' knowledge, the longitudinal relationship between SMA and WA has never previously been investigated, in particular by simultaneously considering the role of temporally stable/trait and temporally varying/occasion-specific factors. Therefore, the findings extend existing research on the relationship between WA and problematic and potentially addictive behaviors related to internet use (i.e., in this case, related to the use of social media sites) (Quinones et al., 2016). Moreover, the associations between WA and SMA at different levels can be understood through different mechanisms.

On the one hand, the significant and positive association was found between SMA and WA trait factors, as well as between state residual factors in the same wave, may be accounted for by the overlap in symptoms as outlined in the CMA (e.g., possibly reflected by the extremely strong association between the trait factors), overlap in risk factors between SMA and WA, and the interaction between work and social media use in everyday life (Gullo et al., 2022; Huang, 2022; Kun et al., 2021; Quinones et al., 2016).

The close association of the two disorders can be explained in several ways. There may be a common vulnerability that characterizes addictive disorders, whether related to personality traits (e.g., negative affectivity, self-esteem) (Dadiotis & Roussos, 2024; Kun et al., 2021), underlying psychopathological factors (e.g., depressive symptoms and compulsivity) (Andreassen, Griffiths, Sinha, Hetland, & Pallesen, 2016; Hussain & Griffiths, 2018), or genetic factors. The genetic factors responsible for WA are not yet known, but for SMA, it is already established that similar genetic differences are present as in other addictive disorders (Vereczkei et al., 2022). Additionally, the presence of Reward Deficiency Syndrome is a common background underlying many addictive disorders (Blum et al., 2000; Kótyuk, Potenza, Blum, & Demetrovics, 2022), and the assumed hypodopaminergic characteristics might be also relevant for SMA and WA. Another interpretation is that excessive and potentially addictive social media use may keep individuals more engaged in the online space, including spending more time on work-related activities (e.g., reading and writing emails, gathering work-related information, communicating at work, etc.). However, the extremely high correlation between the trait factors of SMA and WA might have occurred due to some kind of statistical artefact (e.g., suppressor effects of the trait factors), so caution is needed when interpreting the related findings.

On the other hand, as aforementioned, somewhat conflicting cross-lagged relationships were shown between SMA and WA. SMA negatively predicted subsequent levels of WA, while WA positively predicted subsequent SMA. The former negative relationship may be interpreted in light of the idea that it is difficult to have comorbidity between two BAs given their time-consuming nature (Griffiths, 2018). The negative effect of SMA on WA also contradicts the findings of Quinones et al. (2016). They found that compulsive internet use increased subsequent WA symptoms. According to this, although compulsive internet use in general may be a risk factor for later WA, the potentially addictive use of social media does not appear to be such a risk factor.

A possible explanation for the divergent longitudinal relationships may be that compulsive internet use can be considered as a broader concept that may be more explicitly linked to and covers work-related activities (e.g., reading and writing emails, gathering work-related information), therefore contributing to its role as a risk factor for WA. In contrast, the specific activity of social media use is primarily associated with recreation and personal life (Wang, Jackson, Wang, & Gaskin, 2015), rather than work. The negative relationship between SMA and WA may therefore be accounted for by the possibility that these are competing areas of life in terms of time expenditure (Griffiths, 2018). A plausible explanation for the positive cross-lagged relationship between WA and SMA is that individuals affected by WA are more inclined to use social media, and possibly in a more problematic manner, because their continuous work commitments tie them to their devices. Consequently, they are more likely to begin using social media for work-related purposes. For example, continuous online activity in working may act as a catalyst to promote addictive tendencies initially present related to work to later transmit to social media use.

### Limitations and future directions

The methodological shortcomings of the study should also be acknowledged when interpreting the results. First, the generalizability of the findings to the entire adult population in Hungary is not possible, and conclusions drawn on the longitudinal course of other BAs and the CMA in general are only cautiously recommended. Second, it is expected that the results would differ to some degree if data from the participants who dropped out and/or were excluded from the sample had been considered (e.g., those who worked less than 40 h might show a different pattern of SMA, different work-related and socioeconomic characteristics of those who experienced a negative impact on their employment due to COVID-19 pandemic). Third, the measurement of SMA and WA had several limitations. The influence of social desirability cannot be ruled out following the use of the self-report scales. The symptoms described by the CMA were only briefly assessed by one item and Griffiths (2019) has noted that few scales using CMA as their theoretical basis have items that fully assess each component. Fourth, either potentially relevant symptom dimensions of SMA and WA (not considered by the CMA) or alternative conceptualizations of SMA and WA (e.g., obsessive-compulsive-, or impulsive control disorders) were not included and tested. For both constructs, a generalized approach was used (e.g., different social media platforms and job types could have different symptom characteristics). Fifth, the trait WA factor had very low reliability, therefore cautious interpretation of associations with it is recommended. In addition, the study did not analyze the data regarding potential changes in individuals' employment relationships, workplaces, or responsibilities, which were significant situational factors related to WA during these two years of data collection. Similarly, the research team did not have the opportunity to analyze the moderating effect of COVID-19 epidemic, including its impact on health, social relationships, and work.

Given these limitations, it may be worth investigating the longitudinal structure of CMA in other BAs in the future, and specifically among clinical or high-risk populations (characterized with higher symptom severities), as well as among a sample representative of the entire adult population. It would also be valuable to capture the symptoms of the CMA in more detail using a longer and more intensive longitudinal design. Future studies should consider examining the similarities and differences in the predictors of temporally stable/trait and temporally varying/occasion-specific factors across BAs. It would be useful to conduct future longitudinal studies in which the symptoms of BAs are analyzed by considering a combination of individual and situational factors, such as interactions. Finally, regarding the association between SMA and WA, it would be valuable to investigate which specific social media platforms are more commonly associated with WA and which activities are more typical, as well as to explore the underlying shared motivational factors of social media use and working.

## Conclusions

The present study showed that SMA appears to be approximately equally a trait-like and a state-like construct, whereas WA appears to be very largely state-like among young adults. In light of this, careful consideration is warranted concerning screening for BAs (specifically for SMA and WA) defined within the CMA framework. For both SMA and WA, situational factors have been found to have a significant impact, and it might be therefore desirable to incorporate these as well in the screening process (e.g., assessing recent adverse life events and stressors, changes in job demands). Furthermore, their influence raises concerns regarding the validity of prevalence data for BAs from cross-sectional studies. Therefore, it might be useful to assess risk for BAs multiple times and to consider previous symptom severities to identify individuals with potentially progressive and chronic risk and to exclude those maturing out over time (Gullo et al., 2022; Konkolÿ Thege et al., 2015). From the perspective of the CMA, the results highlight that large differences in the longitudinal development of BAs may be typical, and it would be important to highlight similarities not only at the symptom level but also in the longitudinal symptom course between BAs. In addition, discussions on BAs and the CMA should not only focus on the peripheral vs. central distinction of symptoms (i.e., capacity of separating problematic vs. non-problematic behavior), but also on their longitudinal trait vs. state-like nature (i.e., more trait-like symptoms may indicate chronic addictive behavior).

## Supplementary data

## Footnotes

1 It is important to note that neither SMA nor WA are officially acknowledged disorders in the ICD-11 and DSM-5. Therefore, despite the use of the label 'addiction' for these behaviors, they are not considered clinically diagnosable disorders. Building on the assumptions of the CMA, the label of ‘addiction’ in the present study implies that SMA and WA also share the criteria that may be common to all addictive disorders, regardless of whether it is related to psychoactive substance use or not (Kuss & Griffiths, 2017). It is also important to mention that these constructs are often labeled differently in the literature, for example, workaholism, compulsive working, problematic social media use, problematic social network site use, etc. To ensure terminological consistency, the terms of SMA and WA are used throughout the present study, but it might be possible that different terminology was applied in the cited studies. In addition, to date, there is no clear consensus on the conceptualization of excessive/out-of-control work and social media use as BAs, and other theoretical frameworks also exist that might be relevant (Andreassen & Pallesen, 2016; Moretta, Buodo, Demetrovics, & Potenza, 2022; Morkevičiūtė & Endriulaitienė, 2022; Varona, Muela, & Machimbarrena, 2022).
