# Supplementary material for: Longitudinal trait and state-like differences in the components model of addiction: An illustration through social media addiction and work addiction
Source: J Behav Addict. 2024 Oct 30;13(4):923–37. doi: 10.1556/2006.2024.00055 (PMC11737420; doi:10.1556/2006.2024.00055)
Supplement: Supplementary file 1 [file jba-13-923-s001.pdf]

**Horváth, Zs. et al.: Longitudinal trait and state-like differences in the components model of addiction: An illustration through social media addiction and work addiction**

https://doi.org/10.1556/2006.2024.00055

## Supplementary materials

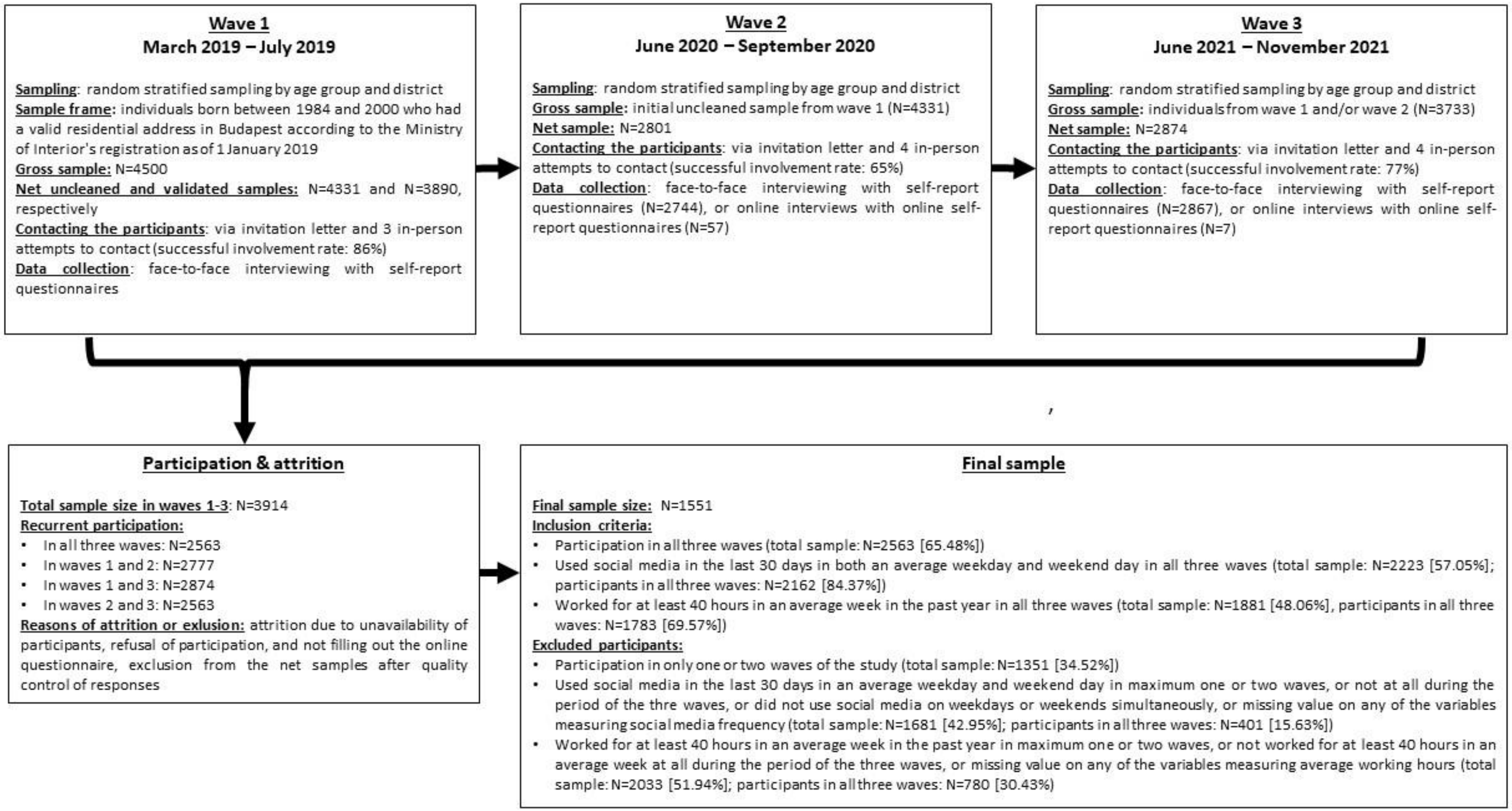


Supplementary Figure S1. Description of the research procedure and inclusion/exclusion criteria

Supplementary Table S1. Descriptive statistics on frequency of social media use and work (N=1551)

| | **Wave 1** | **Wave 2** | **Wave 3** |
|---|---|---|---|
| ***Social media use in the last 30 days in an average weekday N (%)*** | | | |
| Less than an hour | 504 (32.45%) | 573 (36.95%) | 505 (32.53%) |
| 1-2 hours | 690 (44.51%) | 597 (38.51%) | 658 (42.45%) |
| 3-4 hours | 224 (14.47%) | 285 (18.36%) | 379 (24.43%) |
| 5-6 hours | 80 (5.13%) | 79 (5.10%) | 9 (0.59%) |
| 7-8 hours | 36 (2.31%) | 17 (1.08%) | 0 (0.00%) |
| More than 8 hours | 17 (1.10%) | 0 (0.00%) | 0 (0.00%) |
| ***Social media use in the last 30 days in an average weekend day N (%)*** | | | |
| Less than an hour | 430 (27.70%) | 457 (29.49%) | 473 (30.48%) |
| 1-2 hours | 628 (40.48%) | 579 (37.32%) | 658 (42.41%) |
| 3-4 hours | 331 (21.32%) | 351 (22.65%) | 395 (25.44%) |
| 5-6 hours | 94 (6.08%) | 94 (6.04%) | 24 (1.57%) |
| 7-8 hours | 48 (3.08%) | 65 (4.22%) | 1 (0.09%) |
| More than 8 hours | 21 (1.34%) | 4 (0.28%) | 0 (0.00%) |
| ***Working hours in an average week in the past year*** | | | |
| M (SD) | 42.12 (4.30) | 41.74 (4.03) | 41.63 (3.76) |
| Range | 40 – 70 | 40 – 70 | 40 – 70 |

Supplementary Table S2. Description of the invariance models

| | |
|---|---|
| **Latent factor(s) of the model** | A one-factor model was tested related to the Bergen Social Media Addiction Scale (BSMAS) and the Bergen Work Addiction Scale (BWAS). Therefore, these latent factors measured the symptom severities of social media (SMA) and work addiction (WA), respectively. Previous studies supported the one-factor models of the BSMAS and the BWAS [1,2]. |
| **Observed indicators of the model** | The indicator variables of the latent factors of SMA and WA were the six items of the BSMAS and the seven items of the BWAS, respectively. That is, all six items of the BSMAS loaded on a general factor of SMA, and all seven items of the BWAS loaded on a general factor of WA. |
| **Estimation method** | Due to the ordinal nature and skewed distribution of the indicator variables, the models were estimated using the weighted least squares means and variances adjusted (WLSMV) procedure. |
| **Literature background of the tested invariance models** | [3] |
| **Configural invariance model** | The first item of the BSMAS and the BWAS (i.e., salience) was selected as marker items in all three waves, so their factor loadings were fixed at 1.00, while the factor loadings of the other indicator variables were estimated freely. For these marker variables, the thresholds between the first and second response category and between the second and third response category were fixed in equal across measurement occasions. For the other items, the threshold between the first and second response category was fixed in equal across measurement occasions. In the first wave, the residual variances of all observed variables were fixed at 1.00, while in the second and third waves, the residual variances of the observed indicators defining the latent factors were estimated freely. |
| **Metric invariance model** | The factor loadings of each item were fixed in equal over time, and the first item remained as a marker item, so its factor loading was fixed at 1.00. For the marker variable, the thresholds between the first and second response category and between the second and third response category were fixed in equal across measurement occasions. For the other items, the threshold between the first and second response category was fixed in equal across measurement occasions. In the first wave, the residual variances of all variables were fixed at 1.00, while in the second and third waves, the residual variances of the observed indicators were estimated freely. |
| **Scalar invariance model** | The factor loadings of each item were fixed in equal over time, and the first item remained as a marker item, so its factor loading was fixed at 1.00. For each item, the thresholds between response categories were fixed in equal values over time. In the first wave, the residual variances of all variables were fixed at 1.00, while in the second and third waves, the residual variances of the observed indicators were estimated freely. |
| **Residual invariance model** | The factor loadings of each item were fixed in equal over time, and the first item remained as a marker item, so its factor loading was fixed at 1.00. For each item, the thresholds between response categories were fixed in equal values over time. Residual variances of all observed variables were fixed at 1.00. |
| **Latent means** | For each of the invariance models, the mean of the latent factor measured in the first wave were fixed at 0, while in the second and third waves the means of the latent factors were freely estimated. |

| | |
|---|---|
| **Inter-factor and unique residual correlations** | The three latent factors were freely correlated with each other over time. In contrast to the invariance models proposed by Liu et al. [3], the present invariance models did not allow for unique residual correlations of each item with itself over time in any of the invariance models. The reason for this was that the assumption of correlated uniqueness in latent state-trait (LST) models may contribute to a number of statistical and methodological problems, e.g. factors may lose their originally intended meaning or become confounded and therefore cannot be considered as a true LST model, can result in having less parsimonious models, specific variance related to method effects cannot be captured, lack of correlation between method effects may not be an appropriate assumption, and lower levels of reliability of observed indicators may be presented [4]. |

**Cited references:**


1. Bányai, F. *et al.* Problematic Social Media Use: Results from a Large-Scale Nationally Representative Adolescent Sample. *PLOS ONE* **12**, e0169839 (2017).
2. Orosz, G., Dombi, E., Andreassen, C. S., Griffiths, M. D. & Demetrovics, Z. Analyzing Models of Work Addiction: Single Factor and Bi-Factor Models of the Bergen Work Addiction Scale. *Int. J. Ment. Health Addict.* **14**, 662–671 (2016).
3. Liu, Y. *et al.* Testing measurement invariance in longitudinal data with ordered-categorical measures. *Psychol. Methods* **22**, 486–506 (2017).
4. Geiser, C. & Lockhart, G. A comparison of four approaches to account for method effects in latent state–trait analyses. *Psychol. Methods* **17**, 255–283 (2012).

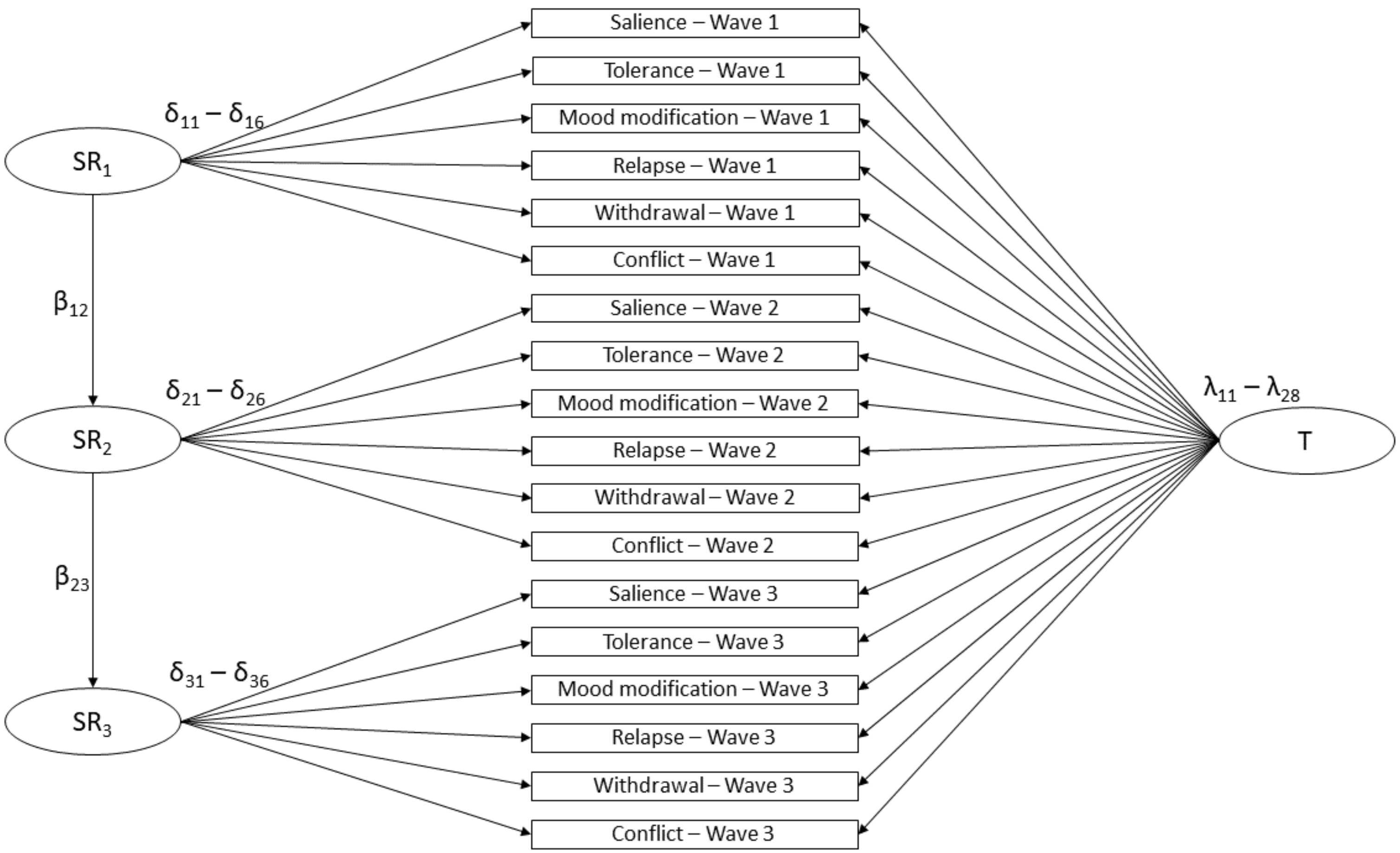


Supplementary Figure S2. Graphical illustration of the latent state-trait (LST) model with one general trait factor and without method factors related to the Bergen Social Media Addiction Scale (BSMAS). $SR_1$-$SR_3$: latent state residual factors. T: latent general trait factor. δ: state residual factor loadings. λ: trait factor loadings. β: autoregressive effects between state residual factors. Salience was a marker item for $SR_1$-$SR_3$, therefore its factor loading (δ) was fixed at 1.00 on $SR_1$-$SR_3$. The factor loadings (δ) of the other items were fixed in equal over time. All factor loadings (λ) on T were fixed at 1.00. For each item, the thresholds between response categories were fixed in equal values over time. Residual variances of all observed variables were fixed at 1.00. The latent means of $SR_1$ and T were fixed at 0.00, while latent means of $SR_2$-$SR_3$ were freely estimated.

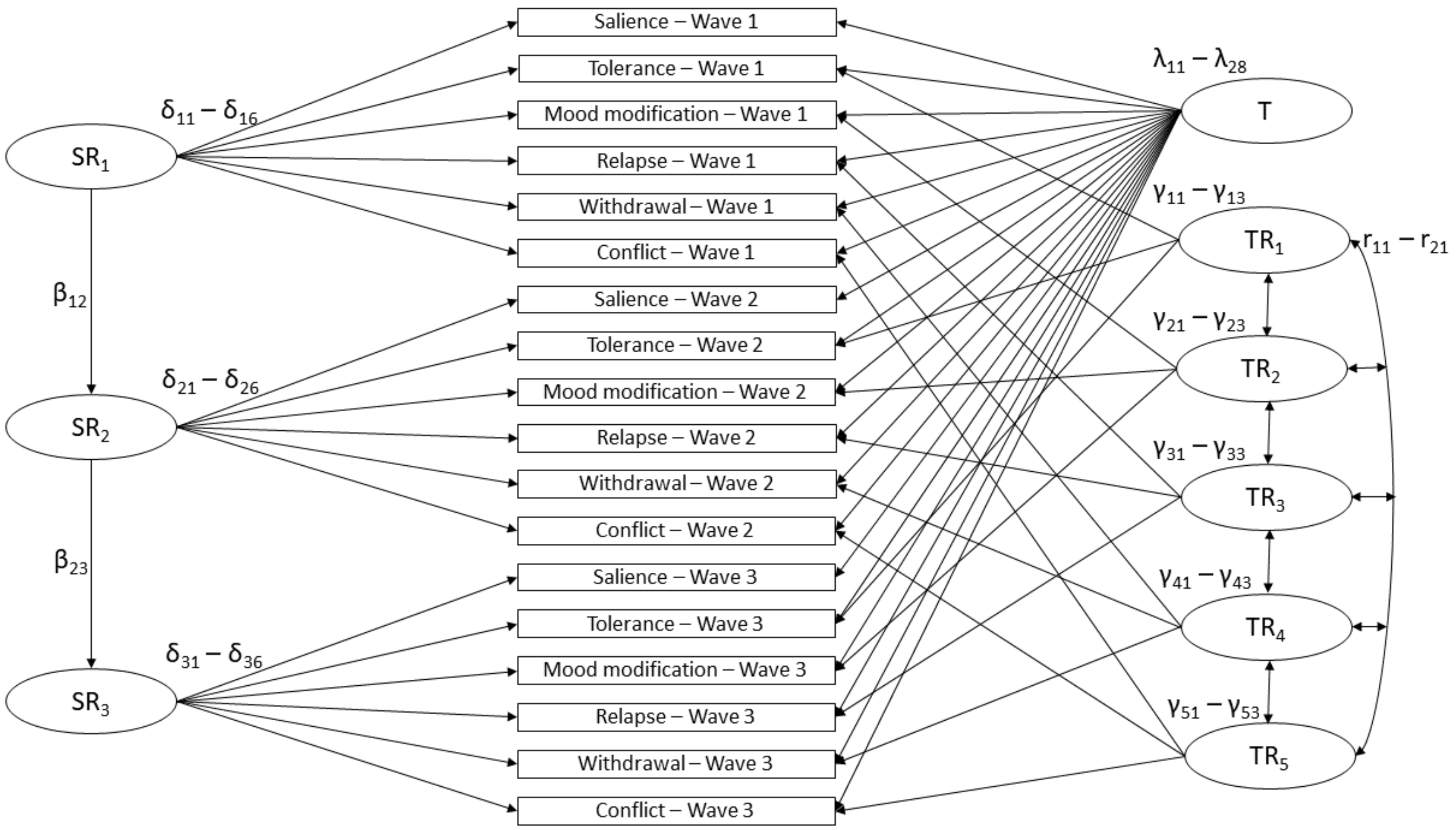


Supplementary Figure S3. Graphical illustration of the latent state-trait (LST) model with M-1 correlated latent method factors (with one general trait factor and five correlated method factors) related to the Bergen Social Media Addiction Scale (BSMAS). $SR_1$-$SR_3$: latent state residual factors. T: latent general trait factor. $TR_1$-$TR_5$: latent indicator specific trait residual factors. δ: state residual factor loadings. λ: trait factor loadings. γ: method factor loadings. β: autoregressive effects between state residual factors. r: correlations between trait factors. Salience was a marker item for $SR_1$-$SR_3$, therefore its factor loading (δ) was fixed at 1.00 on $SR_1$-$SR_3$. The factor loadings (δ) of the other items were fixed in equal over time. All factor loadings (λ, γ) on T, and $TR_1$-$TR_5$ were fixed at 1.00. For each item, the thresholds between response categories were fixed in equal values over time. Residual variances of all observed variables were fixed at 1.00. The latent means of $SR_1$, T, and $TR_1$-$TR_5$ were fixed at 0.00, while latent means of $SR_2$-$SR_3$ were freely estimated.

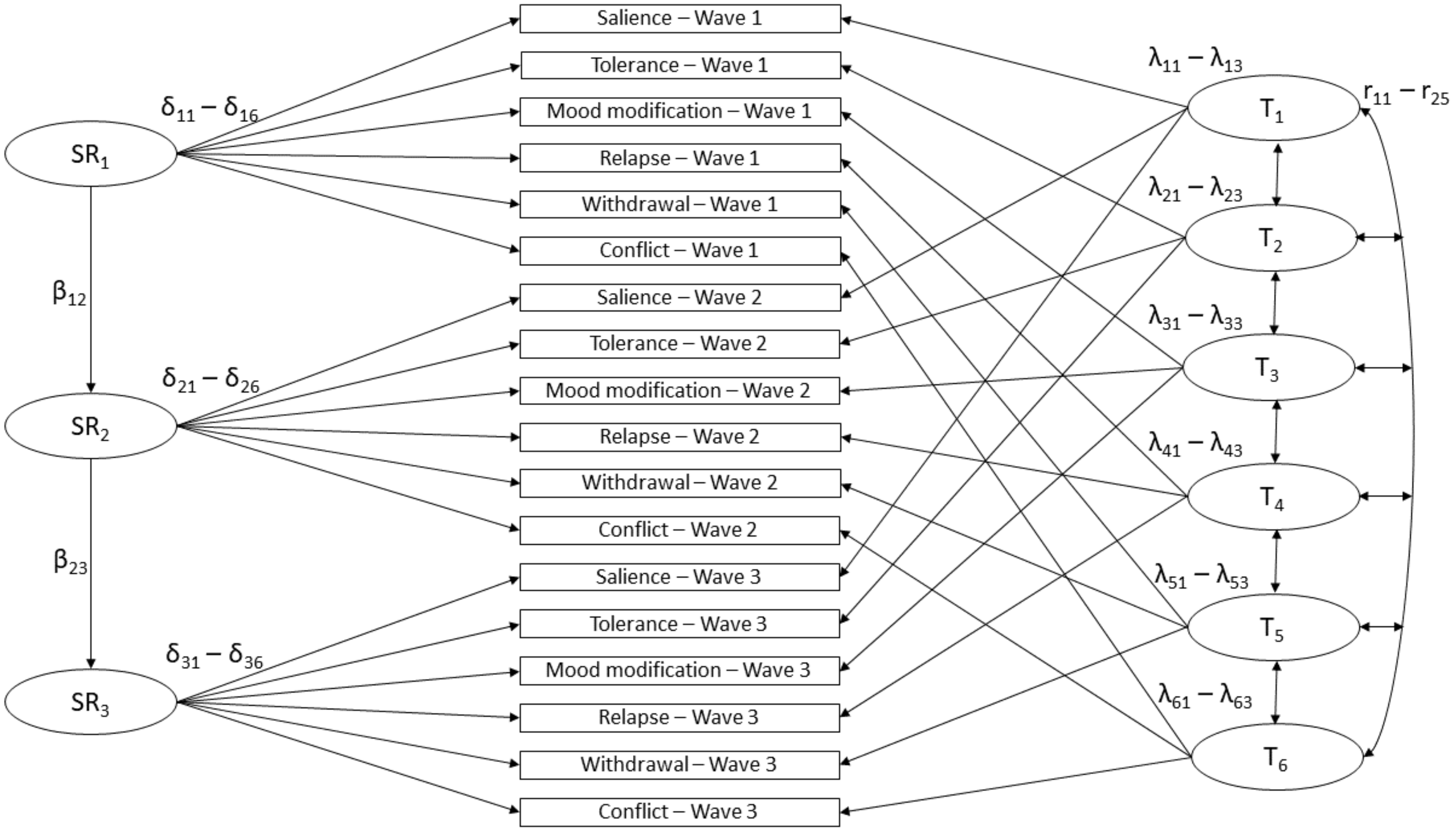


Supplementary Figure S4. Graphical illustration of the latent state-trait (LST) model with six correlated indicator-specific trait factors related to the Bergen Social Media Addiction Scale (BSMAS). $SR_1$-$SR_3$: latent state residual factors. $TR_1$-$TR_6$: latent indicator-specific trait factors. δ: state residual factor loadings. λ: trait factor loadings. β: autoregressive effects between state residual factors. r: correlations between trait factors. Salience was a marker item for $SR_1$-$SR_3$, therefore its factor loading (δ) was fixed at 1.00 on $SR_1$-$SR_3$. The factor loadings (δ) of the other items were fixed in equal over time. All factor loadings (λ) on $T_1$-$T_6$ were fixed at 1.00. For each item, the thresholds between response categories were fixed in equal values over time. Residual variances of all observed variables were fixed at 1.00. The latent means of $SR_1$, and $T_1$-$T_6$ were fixed at 0.00, while latent means of $SR_2$-$SR_3$ were freely estimated.

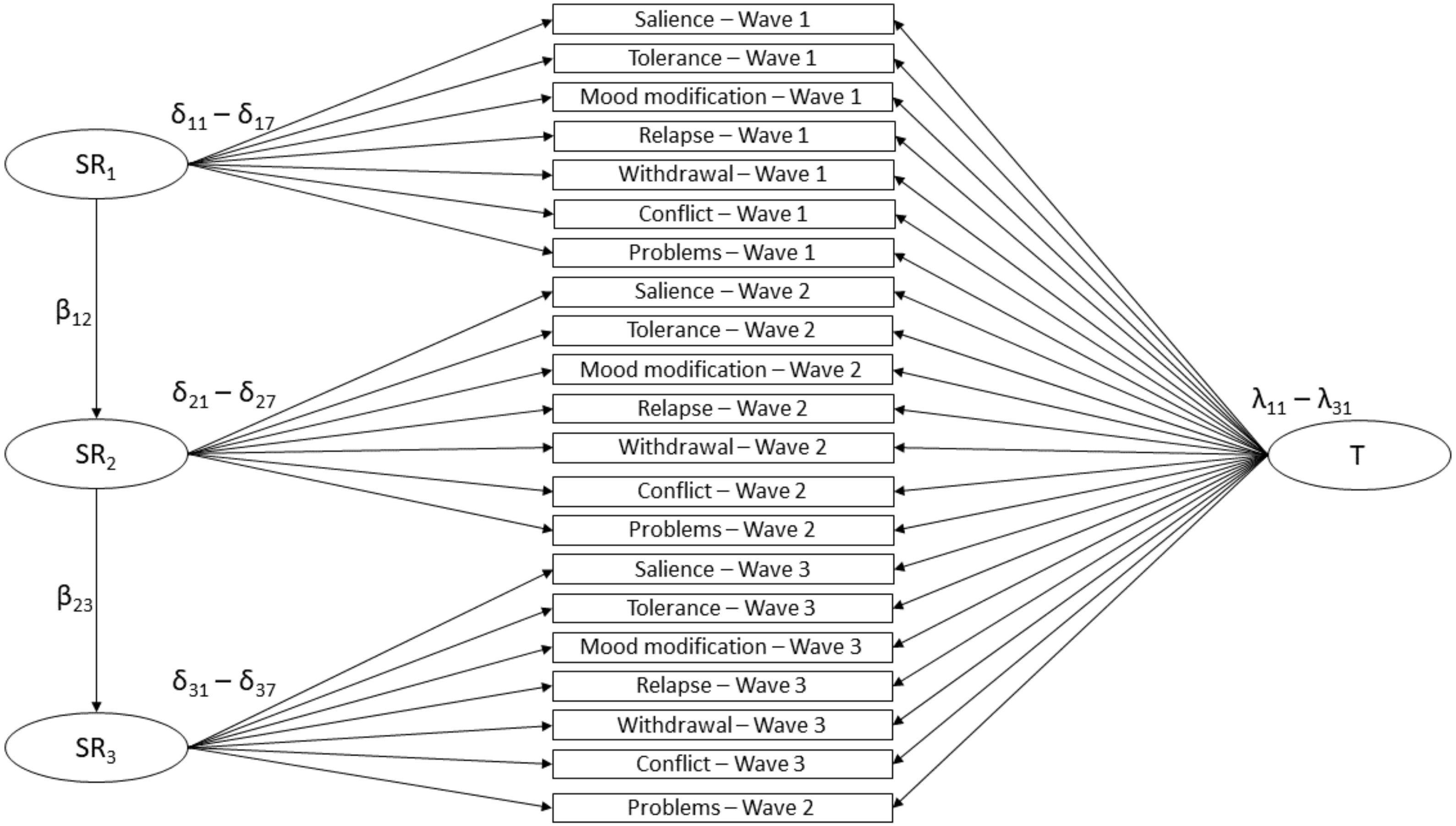


Supplementary Figure S5. Graphical illustration of the latent state-trait (LST) model with one general trait factor and without method factors related to the Bergen Work Addiction Scale (BWAS). $SR_1$-$SR_3$: latent state residual factors. T: latent general trait factor. δ: state residual factor loadings. λ: trait factor loadings. β: autoregressive effects between state residual factors. Salience was a marker item for $SR_1$-$SR_3$, therefore its factor loading (δ) was fixed at 1.00 on $SR_1$-$SR_3$. The factor loadings (δ) of the other items were fixed in equal over time. All factor loadings (λ) on T were fixed at 1.00. For each item, the thresholds between response categories were fixed in equal values over time. The latent means of $SR_1$ and T were fixed at 0.00, while latent means of $SR_2$-$SR_3$ were freely estimated.

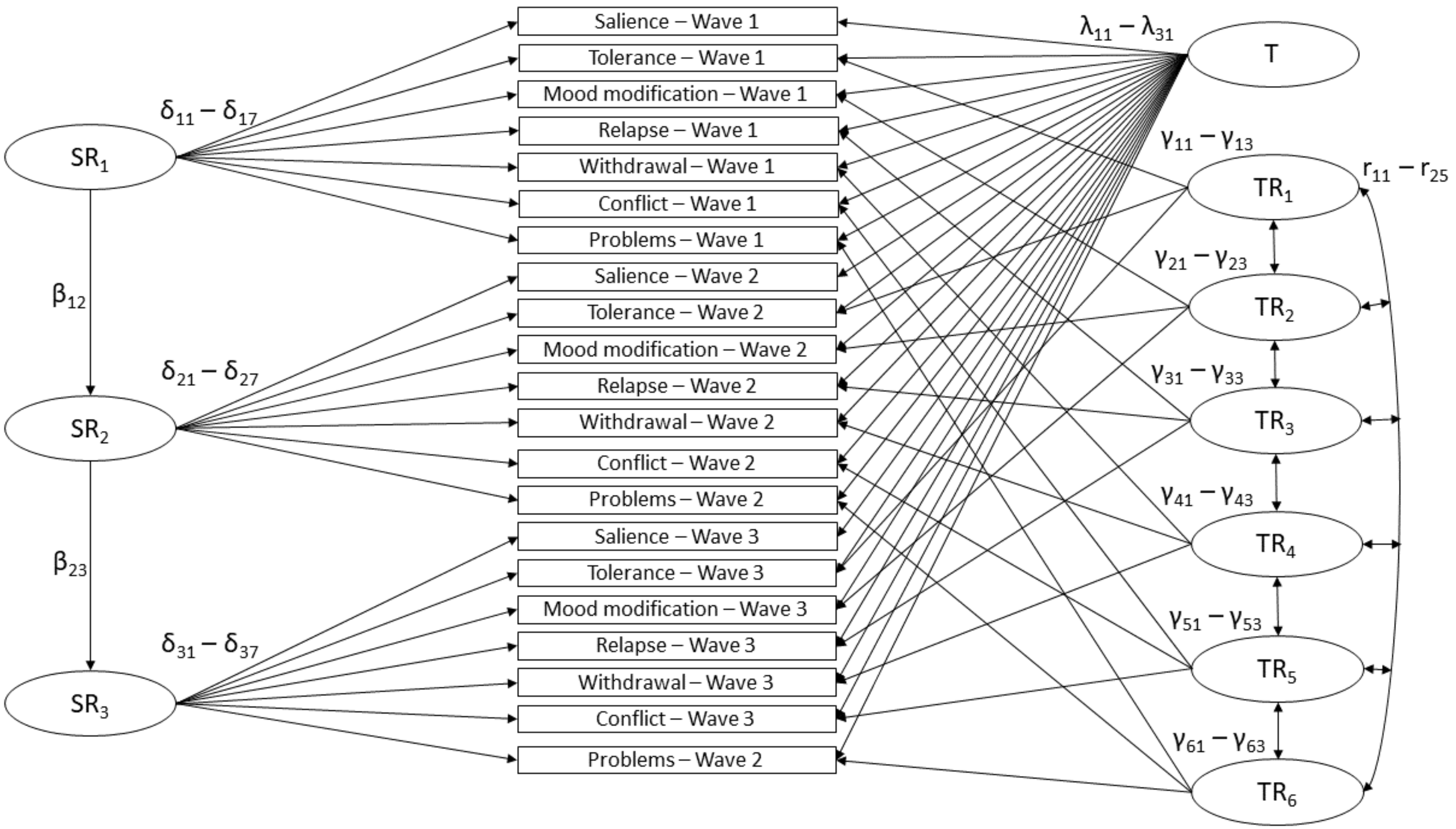


Supplementary Figure S6. Graphical illustration of the latent state-trait (LST) model with M-1 correlated latent method factors (with one general trait factor and six correlated method factors) related to the Bergen Work Addiction Scale (BWAS). $SR_1$-$SR_3$: latent state residual factors. T: latent general trait factor. $TR_1$-$TR_6$: latent indicator specific trait residual factors. δ: state residual factor loadings. λ: trait factor loadings. γ: method factor loadings. β: autoregressive effects between state residual factors. r: correlations between trait factors. Salience was a marker item for $SR_1$-$SR_3$, therefore its factor loading (δ) was fixed at 1.00 on $SR_1$-$SR_3$. The factor loadings (δ) of the other items were fixed in equal over time. All factor loadings (λ, γ) on T, and $TR_1$-$TR_6$ were fixed at 1.00. For each item, the thresholds between response categories were fixed in equal values over time. Residual variances of all observed variables were fixed at 1.00. The latent means of $SR_1$, T, and $TR_1$-$TR_6$ were fixed at 0.00, while latent means of $SR_2$-$SR_3$ were freely estimated.

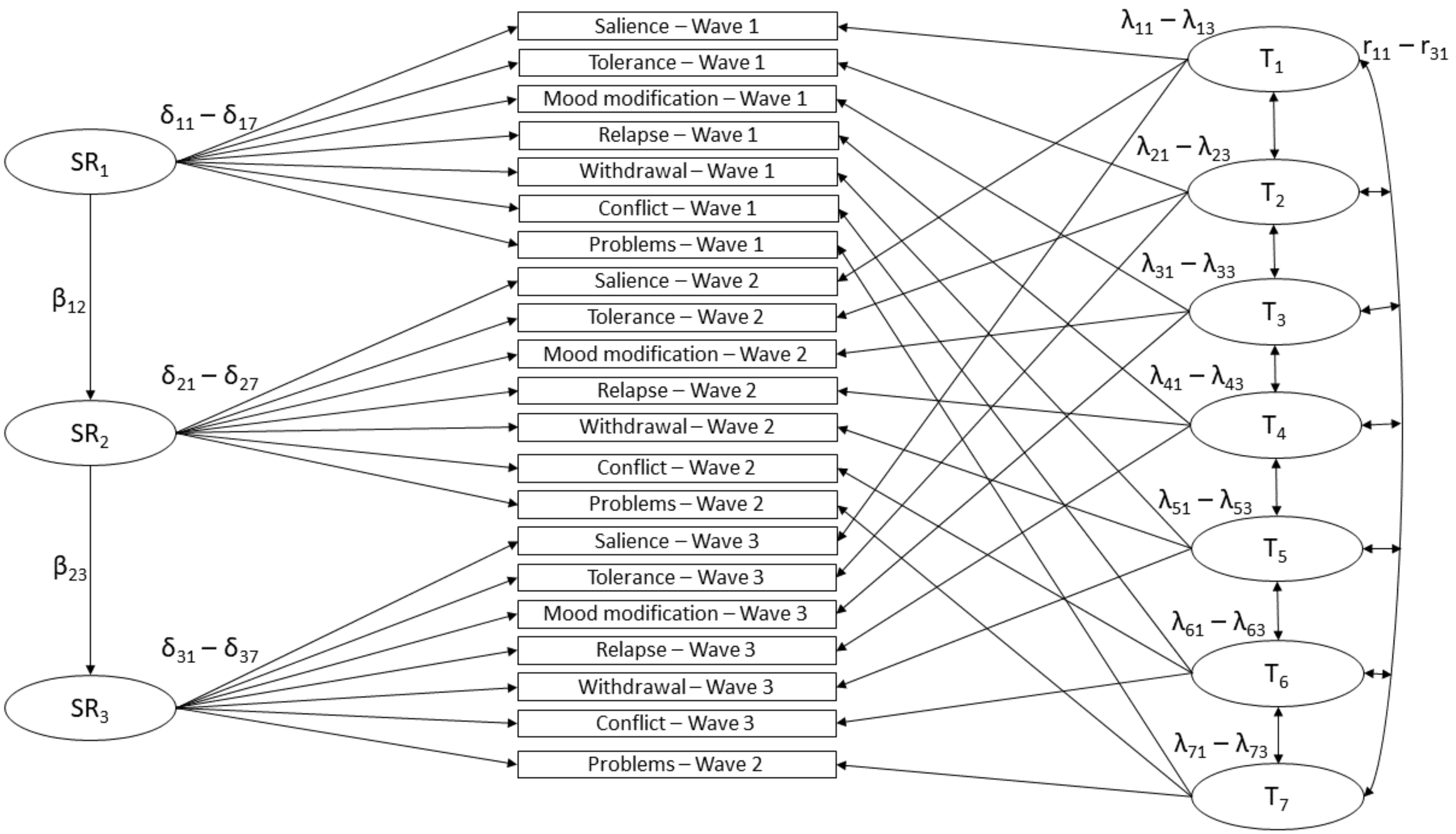


Supplementary Figure S7. Graphical illustration of the latent state-trait (LST) model with seven correlated indicator-specific trait factors related to the Bergen Work Addiction Scale (BWAS). $SR_1$-$SR_3$: latent state residual factors. $TR_1$-$TR_7$: latent indicator-specific trait factors. δ: state residual factor loadings. λ: trait factor loadings. β: autoregressive effects between state residual factors. r: correlations between trait factors. Salience was a marker item for $SR_1$-$SR_3$, therefore its factor loading (δ) was fixed at 1.00 on $SR_1$-$SR_3$. The factor loadings (δ) of the other items were fixed in equal over time. All factor loadings (λ) on $T_1$-$T_7$ were fixed at 1.00. For each item, the thresholds between response categories were fixed in equal values over time. Residual variances of all observed variables were fixed at 1.00. The latent means of $SR_1$, and $T_1$-$T_7$ were fixed at 0.00, while latent means of $SR_2$-$SR_3$ were freely estimated.

Supplementary Table S3. Comparison of the participants included in and excluded from the final sample in terms of social media and work addiction

| | Participants in the final sample (N=1493-1612) M (SD) | Participants excluded from the final sample (N=668-1784) M (SD) | t | Cohen's d |
|---|---|---|---|---|
| Social media addiction – Wave 1 | 7.83 (3.18) | 8.10 (3.49) | 2.40* | 0.08 |
| Social media addiction – Wave 2 | 8.57 (3.56) | 8.12 (3.88) | 3.00** | 0.12 |
| Social media addiction – Wave 3 | 8.05 (3.42) | 7.41 (3.19) | 4,95*** | 0.19 |
| Work addiction – Wave 1 | 4.12 (4.58) | 5.04 (5.79) | 4.77*** | 0.18 |
| Work addiction – Wave 2 | 4.19 (4.77) | 3.83 (5.47) | 1.46 | 0.07 |
| Work addiction – Wave 3 | 5.36 (6.58) | 3.48 (5.52) | 7.36*** | 0.30 |

Notes. Each comparison was performed by using cross-sectional weights. M (SD): mean (standard deviation). t: independent-samples -t-test. Level of significance: *p<.050; **p<.010; ***p<0.001.

Supplementary Table S4. Frequencies of symptom presence and risk for social media addition

| | **Wave 1** | **Wave 2** | **Wave 3** |
|---|---|---|---|
| ***Often or (almost) always present symptoms N (%)*** | | | |
| Salience | 22 (1.49%) | 42 (2.72%) | 52 (3.44%) |
| Tolerance | 26 (1.70%) | 19 (1.21%) | 11 (0.69%) |
| Mood modification | 40 (2.63%) | 49 (3.19%) | 59 (3.83%) |
| Relapse | 30 (2.02%) | 40 (2.64%) | 24 (1.56%) |
| Withdrawal | 24 (1.60%) | 31 (2.01%) | 23 (1.51%) |
| Conflict | 30 (2.02%) | 33 (2.17%) | 24 (1.56%) |
| ***Number of symptoms often or (almost) always present N (%)*** | | | |
| 0 | 1425 (94.56%) | 1419 (92.56%) | 1416 (92.71%) |
| 1 | 42 (2.77%) | 63 (4.08%) | 73 (4.79%) |
| 2 | 16 (1.07%) | 24 (1.58%) | 17 (1.13%) |
| 3 | 7 (0.48%) | 13 (0.85%) | 7 (0.48%) |
| 4 | 10 (0.64%) | 9 (0.57%) | 6 (0.36%) |
| 5 | 5 (0.33%) | 4 (0.28%) | 8 (0.53%) |
| 6 | 2 (0.14%) | 1 (0.08%) | 0 (0.00%) |
| ***Risk for social media addiction N (%)*** | | | |
| Polythetic scoring (≥4 symptoms are present) | 17 (1.12%) | 14 (0.93%) | 14 (0.89%) |
| Monothetic scoring (6 symptoms are present) | 2 (0.14%) | 1 (0.08%) | 0 (0.00%) |

Notes. Missing values are not considered for the calculation of percentages.

Supplementary Table S5. Frequencies of symptom presence and risk for work addition

| | **Wave 1** | **Wave 2** | **Wave 3** |
|---|---|---|---|
| ***More or very typical symptoms N (%)*** | | | |
| Salience | 83 (5.40%) | 117 (7.58%) | 196 (12.72%) |
| Tolerance | 194 (12.56%) | 300 (19.43%) | 176 (11.39%) |
| Mood modification | 41 (2.67%) | 41 (2.69%) | 120 (7.81%) |
| Relapse | 117 (7.57%) | 89 (5.75%) | 124 (8.02%) |
| Withdrawal | 82 (5.30%) | 239 (15.43%) | 119 (7.72%) |
| Conflict | 99 (6.41%) | 84 (5.44%) | 124 (8.07%) |
| Problems | 43 (2.81%) | 45 (2.89%) | 106 (6.86%) |
| ***Number of more or very typical symptoms present N (%)*** | | | |
| 0 | 1196 (77.70%) | 1074 (69.74%) | 1158 (75.37%) |
| 1 | 196 (12.76%) | 202 (13.09%) | 167 (10.90%) |
| 2 | 70 (4.54%) | 182 (11.81%) | 81 (5.26%) |
| 3 | 26 (1.66%) | 28 (1.79%) | 43 (2.79%) |
| 4 | 26 (1.66%) | 31 (2.01%) | 25 (1.66%) |
| 5 | 15 (0.94%) | 14 (0.88%) | 16 (1.02%) |
| 6 | 8 (0.50%) | 6 (0.40%) | 3 (0.17%) |
| 7 | 4 (0.23%) | 4 (0.27%) | 44 (2.84%) |
| ***Risk for work addiction N (%)*** | | | |
| Polythetic scoring (≥4 more or very typical symptoms are present) | 51 (3.34%) | 55 (3.56%) | 87 (5.68%) |
| Monothetic scoring (7 more or very typical symptoms are present) | 4 (0.23%) | 4 (0.27%) | 44 (2.84%) |

Notes. Missing values are not considered for the calculation of percentages.

Supplementary Table S6. Model fit indices of the longitudinal invariance and latent state-trait (LST) models

| | $\chi^2$ (df) | CFI | TLI | RMSEA [90% CI] | Comparison | $\Delta\chi^2$ (df) | ΔCFI | ΔTLI | ΔRMSEA |
|---|---|---|---|---|---|---|---|---|---|
| ***Longitudinal invariance models of the Bergen Social Media Addiction Scale (BSMAS)*** | | | | | | | | | |
| Configural invariance | 548.185 (132) | 0.990 | 0.988 | 0.043 [0.040; 0.047] | | | | | |
| Metric invariance | 572.318 (142) | 0.990 | 0.989 | 0.042 [0.039; 0.046] | Configural vs. Metric | 30.249 (10) | 0.000 | +0.001 | +0.001 |
| Scalar invariance | 648.259 (176) | 0.989 | 0.990 | 0.040 [0.037; 0.043] | Metric vs. Scalar | 115.316 (34) | -0.001 | +0.001 | +0.002 |
| Residual invariance | 688.045 (188) | 0.988 | 0.990 | 0.040 [0.037; 0.043] | Scalar vs. Residual | 72.380 (12) | -0.001 | 0.000 | 0.000 |
| ***Longitudinal invariance models of the Bergen Work Addiction Scale (BWAS)*** | | | | | | | | | |
| Configural invariance | 1283.443 (186) | 0.982 | 0.980 | 0.059 [0.056; 0.062] | | | | | |
| Metric invariance | 1302.276 (198) | 0.982 | 0.981 | 0.058 [0.055; 0.061] | Configural vs. Metric | -[1] | 0.000 | +0.001 | +0.001 |
| Scalar invariance | 1728.887 (238) | 0.976 | 0.979 | 0.061 [0.058; 0.064] | Metric vs. Scalar | 538.400 (40) | -0.006 | -0.002 | -0.003 |
| Residual invariance | 2408.403 (252) | 0.965 | 0.971 | 0.071 [0.069; 0.074] | Scalar vs. Residual | 694.923 (14) | -0.011 | -0.008 | -0.010 |
| ***Latent state-trait (LST) models of the Bergen Social Media Addiction Scale (BSMAS)*** | | | | | | | | | |
| One trait factor without method factors | 1011.248 (188) | 0.980 | 0.984 | 0.051 [0.048; 0.054] | | | | | |
| One trait factor with five correlated method factors[2] | 522.635 (173) | 0.992 | 0.993 | 0.035 [0.031; 0.038] | | | | | |
| Six correlated indicator specific trait factors[2] | 498.436 (168) | 0.992 | 0.993 | 0.034 [0.031; 0.038] | | | | | |
| ***Latent state-trait (LST) models of the Bergen Work Addiction Scale (BWAS)*** | | | | | | | | | |
| One trait factor without method factors | 1773.159 (238) | 0.975 | 0.978 | 0.062 [0.059; 0.065] | | | | | |
| One trait factor with six correlated method factors[2] | 1422.075 (217) | 0.980 | 0.981 | 0.057 [0.055; 0.060] | | | | | |
| Seven correlated indicator specific trait factors[2] | 1417.949 (211) | 0.980 | 0.980 | 0.058 [0.055; 0.061] | | | | | |

Notes. $\chi^2$ (df): chi-square test of model fit (degrees of freedom). CFI: comparative fit index. TLI: Tucker-Lewis index. RMSEA [90% CI]: root mean square error of approximation [90% confidence interval]. $\Delta\chi^2$ (df): chi-square difference test (degrees of freedom). ΔCFI, ΔTLI, ΔRMSEA: differences on the CFI, TLI, RMSEA between the two consecutive invariance models. Positive values for the comparisons indicate improvement for the more restrictive model (with larger degrees of freedom) and negative values show decrease in model fit for the more restrictive model. All $\chi^2$ and $\Delta\chi^2$ are significant at $p<.001$ (two-tailed). [1]The $\Delta\chi^2$ could not be computed by the DIFFTEST option.[2]The model was not considered due to statistical problems: the latent variable covariance matrix was not positive definite due to one or more correlations ≥ 1.00.
